# Anomalies Unveiled: A Fascinating Case Study of Type I Pulmonary Artery-to-Left Atrium Fistula

**DOI:** 10.7759/cureus.64435

**Published:** 2024-07-12

**Authors:** Samreen Shahid, Mehtab Ahmad, Shaad Abqari, Syed Yusuf Masood

**Affiliations:** 1 Radiodiagnosis, Jawaharlal Nehru Medical College, Aligarh Muslim University, Aligarh, IND; 2 Pediatrics, Jawaharlal Nehru Medical College, Aligarh Muslim University, Aligarh, IND

**Keywords:** cardiac computed tomography, pediatric congenital heart disease, cyanotic congenital heart disease, device closure, fistula, left atrium, pulmonary artery

## Abstract

The pulmonary artery-to-left atrium (LA) fistula is one of the rare and unique structural causes of silent cyanosis. This correctable abnormality can be identified by having a high index of clinical suspicion and appropriate investigations using echocardiography and cardiac computed tomography (CT). We report an eight-year-old child who had worsening exertional dyspnea, long-standing central cyanosis, and recurrent infections. A large-sized fistula connecting the right pulmonary artery (RPA) to the LA with all the right- and left-sided pulmonary veins showed normal drainage into the LA, suggesting a type I RPA-to-LA fistula, which was diagnosed on cardiac CT. Percutaneous closure using the occluder device is planned as further management for the patient.

## Introduction

An abnormal communication rarely occurs between the right pulmonary artery (RPA) and the left atrium (LA), known as RPA-to-LA fistula. This is a rare congenital anomaly in which there is direct communication between the RPA or its branches to the LA, referred to as a fistulous connection, causing right-to-left shunting. Only a few case reports have been documented (less than 100) in the literature due to their rarity and difficult diagnosis, and their actual prevalence is unknown [1]. The RPA-to-LA fistula belongs to the category of cyanotic congenital heart disease, with no intracardiac shunts. Though varying degrees of cyanosis are present in different patients, diagnosis is often delayed due to the minimal accompanying symptoms and silent precordium. Many times, late presentations include complications like brain abscesses, clubbing, hyperviscosity syndrome, and systemic embolization in the late teens.

Clinical diagnosis is often difficult because the ECG and chest X-ray are not helpful in the majority of cases. Thus, early diagnosis is crucial for planning management and preventing further complications and hence requires a strong degree of suspicion in the initial phases of disease.

The management of such cases is still evolving. Both the transcatheter and surgical methods have been described; the latter is less preferable. The age of the patient, the size of the defect, associated anomalies or complications, and the availability of expertise are the major determinants in planning treatment.

## Case presentation

An eight-year-old male child presenting with exertional dyspnea, recurrent fever, cyanosis of the lips and fingers, and chest pain following everyday physical activity was admitted to the department of pediatrics in a tertiary care hospital; however, no hemoptysis, squatting phenomena, seizures, nocturnal sweats, or weight loss were present. There was no significant family history. Upon admission, physical examination revealed cyanosis of the lips and fingers and clubbing with a resting oxygen saturation of 68% on room air, a resting blood pressure of 80/50 mmHg, a heart rate of 120 beats per minute, and a regular breathing rhythm. The cardiovascular examination revealed a silent precordium with normal heart sounds (S1 and S2) on auscultation. The results of other systemic examinations were normal, and symmetric peripheral pulses of the bilateral upper and lower limbs were recorded. The level of hemoglobin was 14.6 g/dL. ECG revealed normal sinus rhythm, rate, and no axis deviation.

Imaging findings

**Figure 1:**
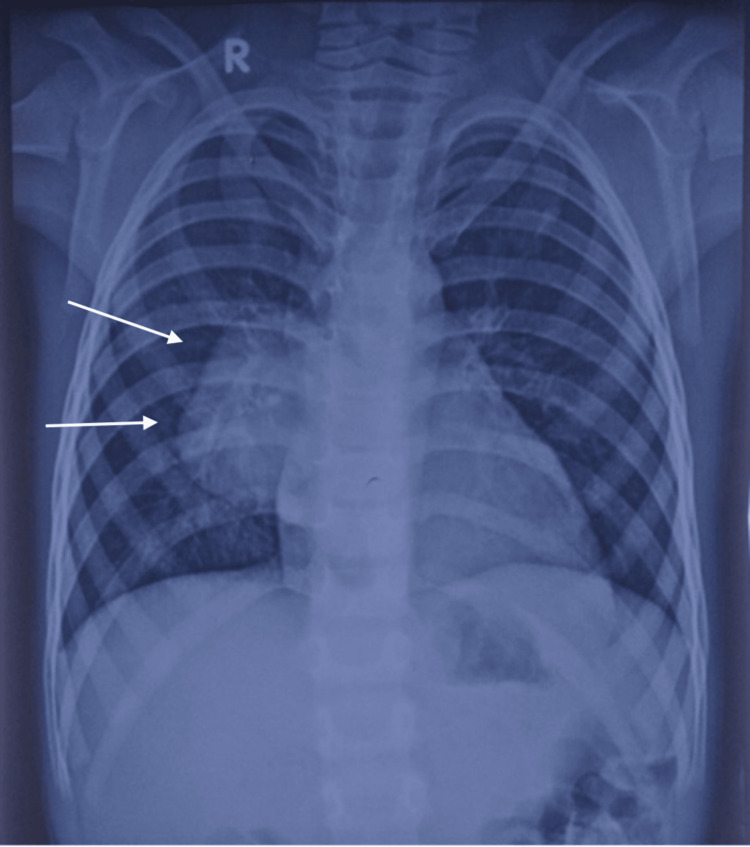
Chest X-ray PA view demonstrating a crescentic radio-opacity in the right para-cardiac region with normal bilateral lung fields (white arrows). PA: posteroanterior

The chest X-ray (posteroanterior view) revealed a crescentic radio-opacity in the right para-cardiac region with normal bilateral lung fields (Figure 1).

On transthoracic echocardiography, situs solitus and levocardia were noted, with an abnormally enlarged LA and an outpouching in the posterosuperior aspect on the right side showing an abnormal color flow on the color Doppler. There were normal ejection fraction, normal relations of great arteries with a left-sided aortic arch, and no coarctation of the aorta. Intact interatrial or interventricular septa were documented (Video 1 and Video 2). The echocardiography was self-explanatory, and so cardiac computed tomography (CT) was performed directly for the confirmation of the diagnosis.

**Video 1:** Echocardiography showing a dilated sac-like structure in the posterosuperior aspect on the right side of the heart showing color flow on Doppler.

**Video 2:** Echocardiography apical four-chamber view showing the dilated aneurysmal sac appearing in the posterosuperior aspect of the left atrium (communicating with the left atrium=shunting of blood).

**Figure 2:**
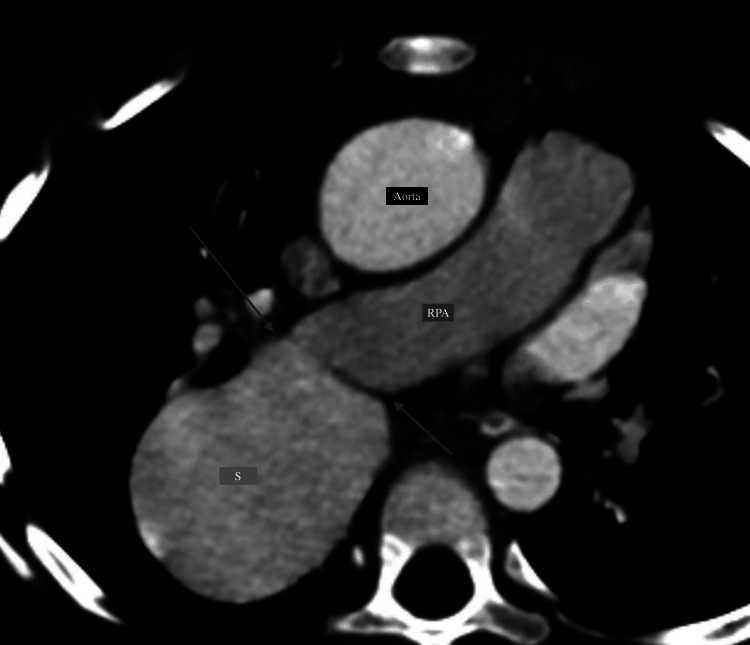
CT reformatted images (axial section) showing dilated aneurysmal sac (labeled as "S") communicating with the descending branch of the right pulmonary artery (labeled as "RPA"). The site of communication between the pulmonary artery and sac is demonstrated by the arrows. This sac is further communicating with the left atrium (shown in Figure 3).

**Figure 3:**
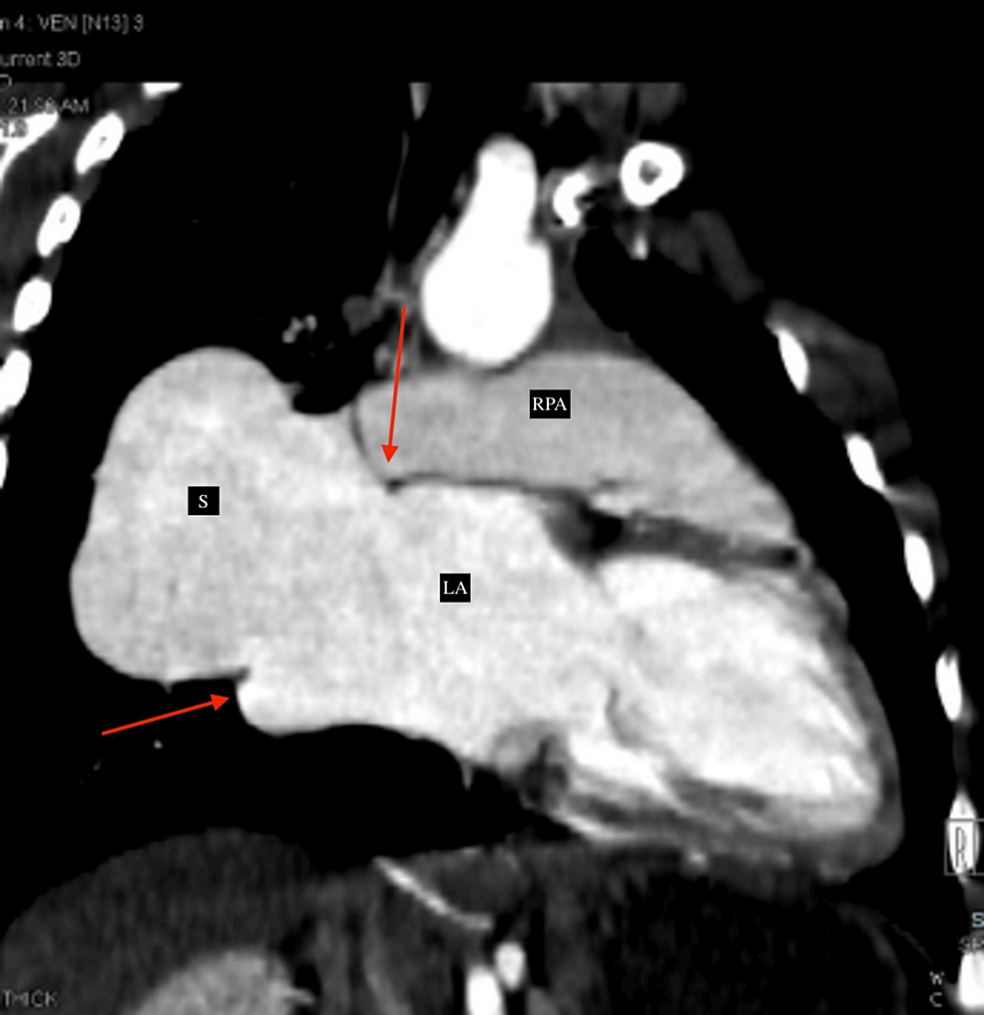
CT reformatted image (coronal section) demonstrating the dilated aneurysmal sac (labeled as "S") draining into the left atrium (labeled as "LA"). The site of communication between the sac and the left atrium is marked by red arrows). RPA: right pulmonary artery

Cardiac CT was performed using a non-gated CT technique using a 128-slice CT machine with a contrast dosage of 1.5 ml/kg, given at a rate of 2.2 ml/sec at 80 kV tube voltage. The findings exhibited a dilated aneurysmal sac-like outpouching (~45×35 mm) on the right side of the LA, which showed direct communication with the descending branch of the RPA (Figure 2) and the opening of the dilated sac being noted into the LA (Figure 3).

**Figure 4:**
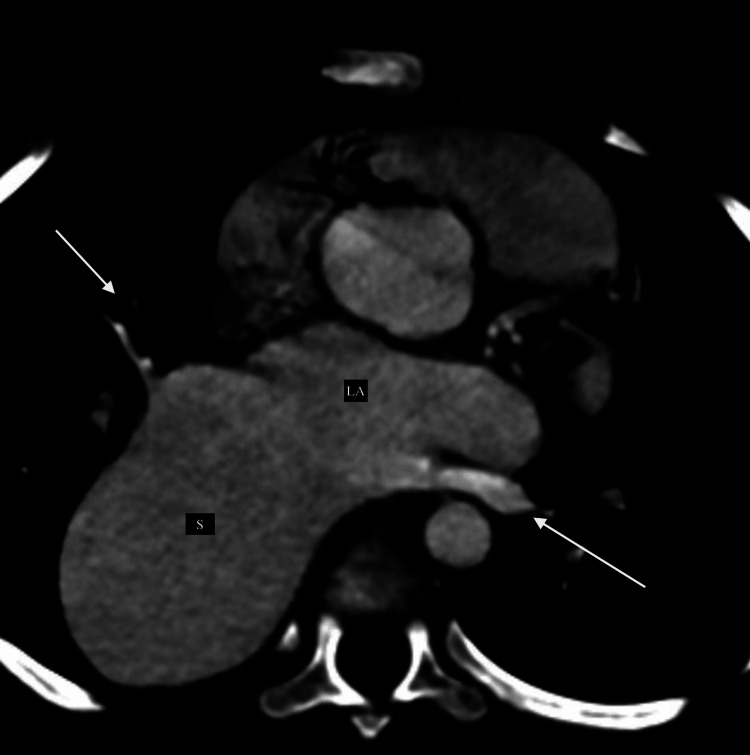
Axial CT reformatted images showing the normal drainage of pulmonary veins into the left atrium (shown by arrows). S: dilated aneurysmal sac: LA: left atrium

All the right and left pulmonary veins drained normally into the LA (as shown in Figure 4).

**Figure 5:**
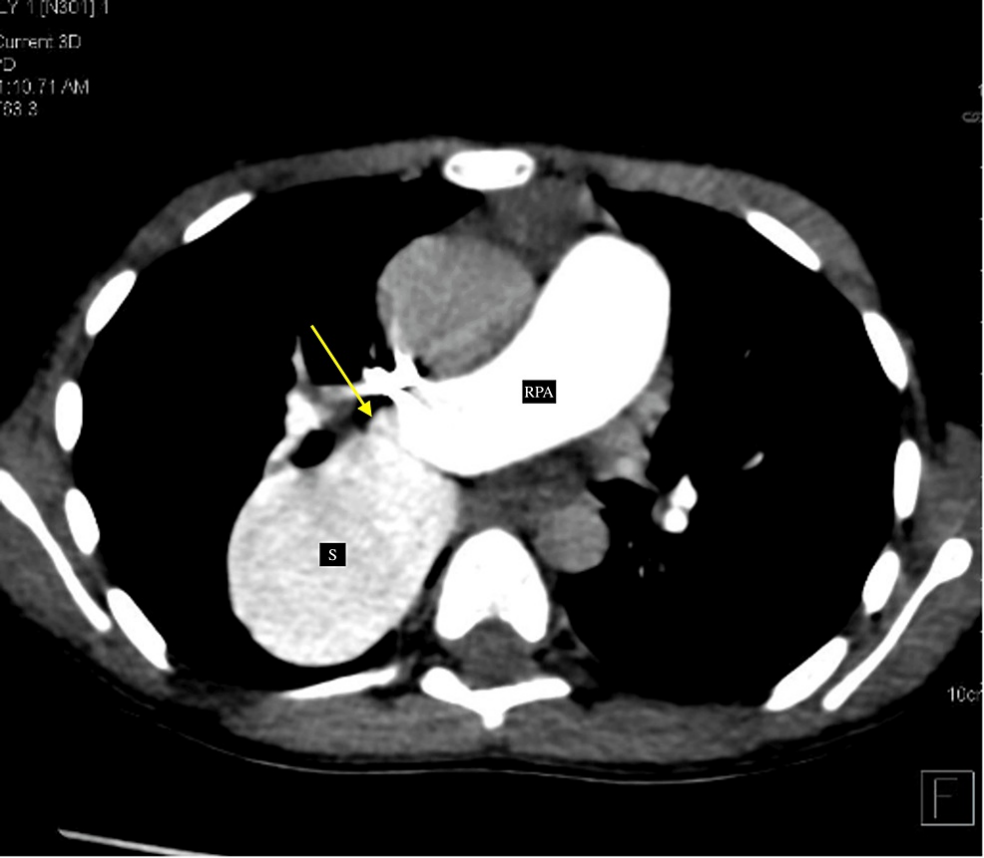
Axial CT reformatted image demonstrating the jet of contrast (yellow arrow) from the RPA in the pulmonary arterial phase into the dilated aneurysmal sac (labeled as "S"). The sac is getting filled in the pulmonary arterial phase, and thus, contrast reaches the left atrium even before the contrast opacification of pulmonary veins. RPA: right pulmonary artery

The sac was getting filled by the contrast during the pulmonary arterial phase and ultimately into the LA even before the contrast opacification of pulmonary veins, therefore suggesting pulmonary artery-to-LA fistula formation (type I), causing a right-to-left shunting (Figure 5).

**Figure 6:**
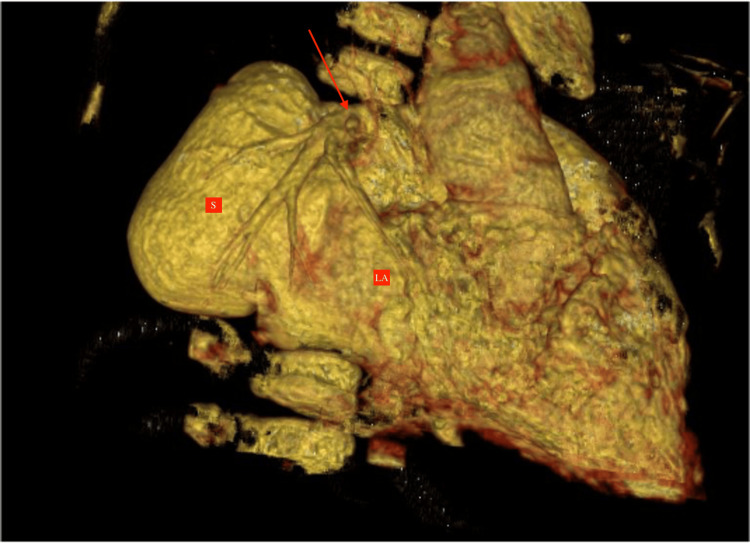
VRT image in the coronal section showing the opening of the dilated aneurysmal sac (labeled as "S") into the left atrium (labeled as "LA"). The red arrow shows the drainage of the pulmonary vein into the left atrium. VRT: virtual reformatted

**Figure 7:**
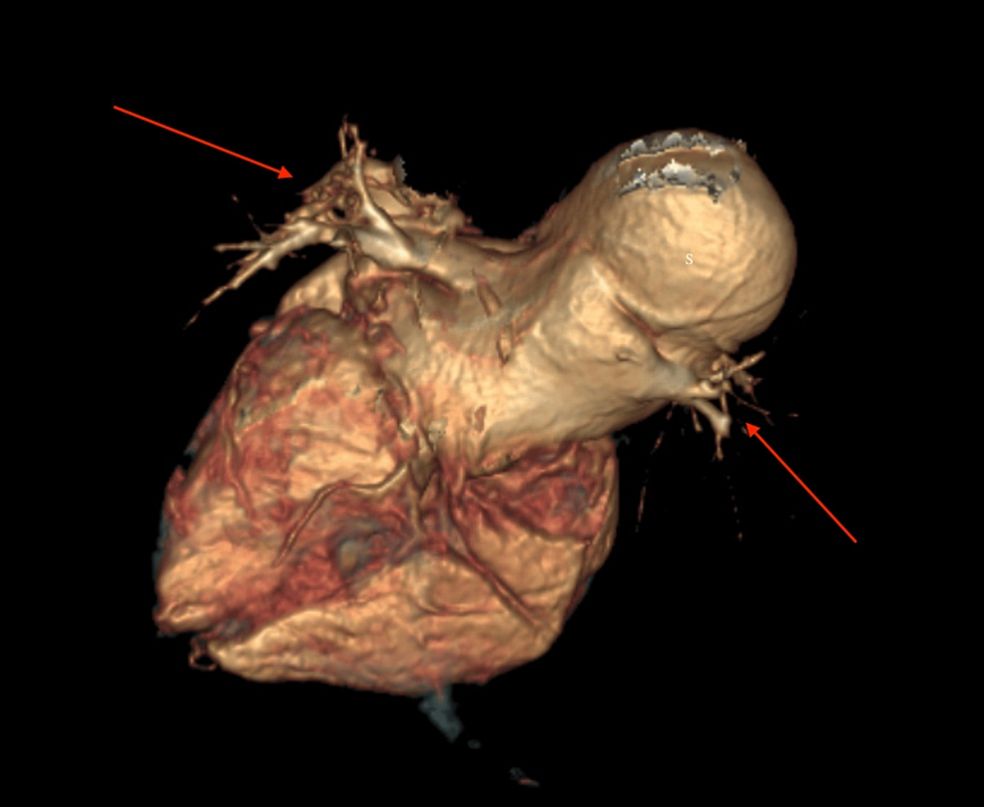
VRT image from the posterosuperior aspect of the heart representing the dilated sac (labeled as "S"), which is opening into the left atrium. Red arrows demonstrate the normal drainage of the pulmonary veins into the left atrium. VRT: virtual reformatted

Normal atrioventricular or ventriculoarterial concordance was noted. The entire right lung's vascularization was normal, and main pulmonary artery (MPA) and RPA branching patterns were normal. No agenesis of the lobe was noted (virtual reformatted images, Figure 6 and Figure 7).

## Discussion

RPA-to-LA fistulas are age-neutral and predominantly affect men (3:1) [2]. About 20% are diagnosed before the age of 10 and 70% before the age of 20. The two main symptoms are exertional dyspnea and central cyanosis [3]. Symptom severity is correlated with the size of the right-to-left shunting. In the early neonatal stages, larger shunts are often present with severe heart failure. Large shunts that are left untreated lead to heart failure, pulmonary edema, and pulmonary hypertension in later life. They cause exertional dyspnea, clubbing, polycythemia, and central cyanosis by acting as direct right-to-left shunts. Avoiding the pulmonary filtering process also allows germs and emboli to enter the bloodstream, which increases the risk of stroke, cerebral abscess, and cerebral and systemic emboli.

Since the RPA-to-LA fistula is neither the primary nor the most frequent cause of the aforementioned symptoms, making a clinical diagnosis of it is extremely difficult [4]. Though echocardiography reveals an abnormally enlarged LA with a dilated sac, it can raise suspicion for the diagnosis. However, RPA-to-LA fistulas and related aneurysms are frequently not visible and are still challenging to diagnose in the initial stages or in cases of smaller fistulas in echocardiography. A contrast echocardiogram can show an aneurysm's early filling and raise the suspicion of abnormal communication. Echocardiography, however, may occasionally fail to identify the anomaly and only reveal turbulence and shunts. The gold standard for confirmation is selective catheter angiography [5]. However, because it is an invasive procedure, it might not always be possible or available and can delay diagnosis and decision-making for management. A more accessible, non-invasive, and precise diagnostic method is CT pulmonary angiography for timely diagnosis and appropriate treatment. CT offers details about the exact size, location, tortuosity, and type of fistula, thereby providing sufficient aid, as they serve as important determinants in management.

Four types based on the communication and presence or absence of pulmonary veins have been described [6]. In category I, an abnormal vessel with normal pulmonary venous connections into the LA drains into the LA from the posterior part of the proximal RPA. In category II, an abnormal vessel joins the RPA's lower lobe branch and empties into the LA at the location of the missing right inferior pulmonary vein. The vessel is aneurysmal close to where it connects to the LA. In category III, the aneurysmal pouch situated between the LA and RPA receives drainage from all pulmonary veins. In category IV, a proximal pulmonary arteriovenous fistula joins the LA. The left-sided pulmonary veins enter the LA directly, and the right-sided pulmonary veins connect the fistulous tract [7].

We have discussed a type I RPA-to-LA fistula case and emphasized the significance of cardiac CT in establishing the diagnosis of this condition. The RPA-to-LA fistula (type I) between the LA and the descending branch of the RPA, leading to the formation of a dilated aneurysmal sac, was the final diagnosis in our patient. Normal drainage of all four pulmonary veins into the LA was noted.

They can be treated with a transcatheter or surgical closure, depending on the size and viability of the fistula [8]. The transcatheter approach includes the use of device occluder devices or atrial septal defect (ASD) or ventricular septal defect (VSD) closure devices, as shown by Singhi et al. [9]. The benefits of catheter-guided procedures include a quicker recovery and fewer procedural and postprocedural problems [10]. In our case, catheter-based device closure of the shunt is being planned and discussed with the family of the patient.

Selecting the right management technique and incision approach is made easier by accurate anatomical identification through CT [11].

## Conclusions

The RPA-to-LA fistula (type I) between the descending branch of the RPA and the LA, leading to the formation of a dilated aneurysmal sac resulting in a right-to-left shunt, was the final diagnosis in this patient. It is a rare cyanotic congenital heart defect that typically manifests as central cyanosis and exertional dyspnea in childhood with no other obvious clinical signs or symptoms, thus making the diagnosis challenging at an early age and hence requiring a strong index of suspicion on echocardiography, which can further be confirmed on CT pulmonary angiography, using that early left atrial opacification (before the opacification of pulmonary veins) due to fistulous communication between the pulmonary artery and the LA can be demonstrated. Management is still evolving and involves correction via transcatheter or surgical closure approaches, which are available for treatment; however, surgical methods are less preferred. To sum up, this case study highlights the significance of the collaborative effort of a pediatrician, radiologist, and surgeon during the diagnosis and management process because the condition is uncommon and needs to be detected early to prevent systemic complications.
